# No association between the Ser9Gly polymorphism of the dopamine receptor D3 gene and schizophrenia: a meta-analysis of family-based association studies

**DOI:** 10.1186/s12881-020-01018-w

**Published:** 2020-04-21

**Authors:** Xiao-na Li, Ji-long Zheng, Xiao-han Wei, Bao-jie Wang, Jun Yao

**Affiliations:** 1 School of Forensic Medicine, China Medical University, No. 77, Puhe Road, Shenyang North New Area, Shenyang, Liaoning Province 110122 People’s Republic of China; 2 Department of Forensic Medicine, Criminal Investigation Police University of China, Shenyang, Liaoning 110035 People’s Republic of China; 3 School of Fundamental Sciences, China Medical University, No. 77, Puhe Road, Shenyang North New Area, Shenyang, Liaoning Province 110122 People’s Republic of China

**Keywords:** Dopamine receptor D3, Schizophrenia, Meta-analysis, Family study

## Abstract

**Background:**

Previous studies found that Ser9Gly (rs6280) might be involved in the occurrence of schizophrenia. However, no consist conclusion has yet been achieved. Compared to the case-control study, the family-based study took into account stratification bias. Thus, we conducted a meta-analysis of family-based studies to measure a pooled effect size of the association between Ser9Gly and the risk of schizophrenia.

**Methods:**

The relevant family-based studies were screened using the electronic databases by the inclusion criteria. Odds ratios (ORs) and 95% confidence intervals (CIs) were used to measure the correction between Ser9Gly polymorphism and schizophrenia susceptibility. Subgroup analysis was performed by stratification of ethnicity (i.e., East Asian, Caucasian, and other populations). Additionally, publication bias was evaluated by the funnel plot.

**Results:**

After literature searching, a total of 13 family-based association studies were included, which contained 11 transmission disequilibrium test (TDT) studies with 1219 informative meiosis and 5 haplotype-based haplotype relative risk (HRR) studies. No statistical significance of the heterogeneity was detected in TDT and HRR studies. Thus, the pooled effect size was calculated under the fixed effect model. The results found that the association was significantly protective in East Asian in TDT studies (204 informative meiosis, OR = 0.744, 95% CI = 0.564–0.980, Z-value = − 2.104, *p* = 0.035).

**Conclusions:**

The meta-analysis based on the family study found a protective association of Ser9Gly in East Asian. In future, large sample molecular epidemiology studies are needed to validate our findings.

## Background

Schizophrenia is a complex mental disorder with the incidence rate of about 1% in the word. Genetic and environmental factors are involved in its pathogenesis [1]. According to the report, heritability of schizophrenia is as high as 80% [2]. So far, there has been no consist outcome regarding the etiology of this mental disorder [3, 4]. Recently, the studies have reported that the dysfunction of dopaminergic neurotransmitter may be involved in the development of schizophrenia [5–8]. Therefore, the genes participating in dopaminergic metabolism are the underlying susceptible genes in this disease.

Dopamine receptor D3 (*DRD3*) is localized to the limbic areas of the brain and are associated with cognitive, emotional, and endocrine functions [9–12]. It is encoded by *DRD3* gene. Ser9Gly variant (rs6280) is a functional polymorphic site in the first exon of *DRD3* gene, which corresponds to a serine to glycine amino acid substitution at position 9 in the extracellular N-terminal domain of *DRD3* [13]. This variation can have a higher affinity for dopamine and amplify dopamine intracellular signaling [14]. Presently, Ser9Gly polymorphism has been reported to be related to neurological and psychiatric disorders [15–18]. For the association between Ser9Gly and schizophrenia, there are still conflicting results without a consistent conclusion [19–23]. These contradictory results may be due to small sample size, inclusion of various genetic backgrounds, and other potential confounding bias [24].

Meta-analyses can merge the various homogeneity studies and arrive at a comprehensive result [25, 26]. Since 1998, the meta-analysis have been conducted to assess the association between Ser9Gly SNP and schizophrenia risk [27–33]. However, all of the pooled results were based on the case-control studies, but not the family-based studies. The family-based studies are more powerful to detect risk factors of schizophrenia, considering that the ability to exploit the cosegregation of variants with schizophrenia within families helps distinguish causal from noncausal factors [34]. Therefore, we carried out a meta-analysis of family-based association studies to better evaluate the relationship between *DRD3* Ser9Gly SNP and the risk of schizophrenia.

## Methods

### Literature search

Three online electronic English databases (Medline, Embase, and Web of Science) and one online Chinese CNKI database were searched using the following key words: “*DRD3”*, “dopamine receptor 3”, “dopamine D3 receptor”, “dopamine receptor D3”, “schizophrenia”, and “Ser9Gly”. Additionally, the other possible studies were screened and retrieved by the reference lists of the included articles and the available reviews.

### Inclusion criteria

**Fig. 1:**
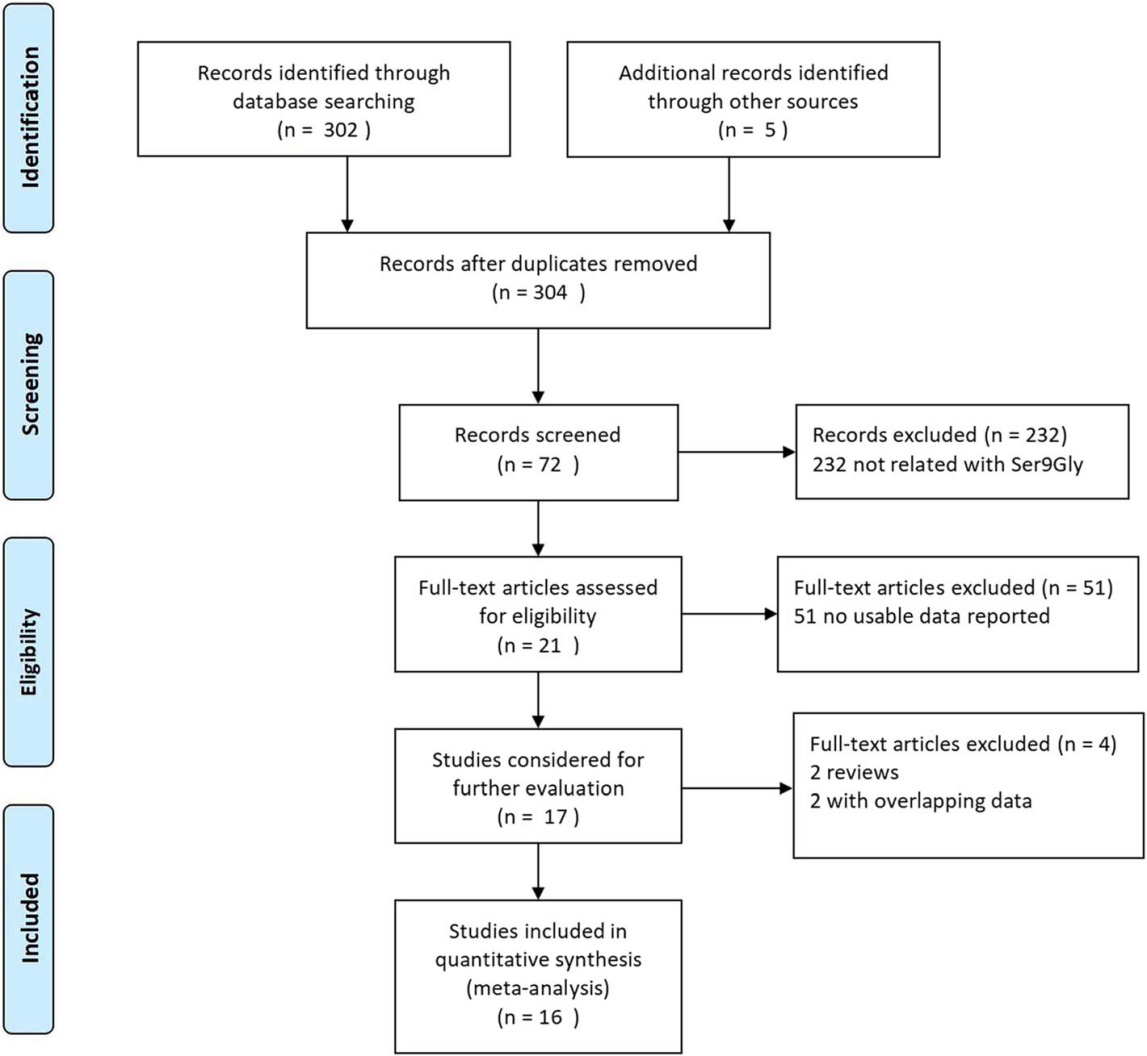
The search flow diagram

The studies reporting Ser9Gly polymorhism were included after meeting the following criteria: (1) family-based design (original transmission disequilibrium test (TDT) [35] or haplotype-based haplotype relative risk (HRR) [36]; (2) original data, or available data to pool an effect size. Finally, we collected 13 articles and the flow diagram of the literature search process was showed in Fig. 1.

### Data extraction

The data extraction was independently conducted by two authors (XNL and BJW). Information collected included last name of first author, year of publication, country, ethnicity of study population, diagnostic criteria for schizophrenia, and numbers of transmissions.

### Meta-analytic methods

The meta-analysis of the family-based association studies was divided into two parts: TDT and HRR. For the TDT study, every included study provided a 2-by-2 transmission disequilibrium table that classifies heterozygous parental alleles (informative meioses) based on the passing status (Ser9 allele passed to the schizophrenic offspring) and data type (the number of observed passing vs. the number of theoretic passing) [37]. For one informative meiosis, the expected transmitted number that the allele is transmitted from heterozygous parents to the proband is 0.5 and the expected untransmitted number that the allele is not transmitted from heterozygous parents to proband is also 0.5. For the HRR studies, every included study provided a 2-by-2 HRR table that classifies parental alleles by type of allele (Ser9 or Gly9) and passing status (passed to the offspring with schizophrenia or not) [37].

The Q test was used to measure the heterogeneity among the included studies and *P* < 0.05 indicated the presence of heterogeneity [38, 39]. Additionally, I^2^ was calculated to quantify the apparent inconsistency and its conventional interpretation for the existed heterogeneity was low (< 25%), moderate (approximately 50%), and high (> 75%) [40]. When there was the existing of heterogeneity (*p* < 0.05 and/or I^2^ > 50%), a random effect model was selelcted; otherwise, a fixed effect model was selected using the Mantel and Haenszel method [38, 41].

For the pooled analysis, odds ratios (ORs) and 95% confidence intervals (CIs) were calculated to quantify the association in the two-by-two Tables. *P* > 0.05 indicated the absence of statistical significance, and *P* < 0.05 indicated statistical significance. When P < 0.05, OR < 1 meant the variation as a protective factor, and OR > 1 meant the variation as a risk factor. Pooled calculations of ORs were obtained and compared with the controls (observed transmission vs. expected transmission for TDT study or transmitted vs. untransmitted for HRR study) using test statistic z and 95% CIs. Moreover, subgroup analysis were conducted by ethnicity (i.e., East Asian, Caucasian, and other populations) and diagnostic criteria (i.e., DSM-III-R, DSM-IV, and CCMD-III). In addition, the funnel plot was generated to evaluate publication bias according to the previous study [37].

All the statistical calculations of the meta-analysis were performed by Comprehensive Meta Analysis V2 software (Biostat, Englewood, NJ, USA).

## Results

A total of 13 articles were identified by database searches, which included 16 studies [27, 42–53]. Among them, 11 studies were for TDT and 5 studies were for HRR.

**Fig. 2:**
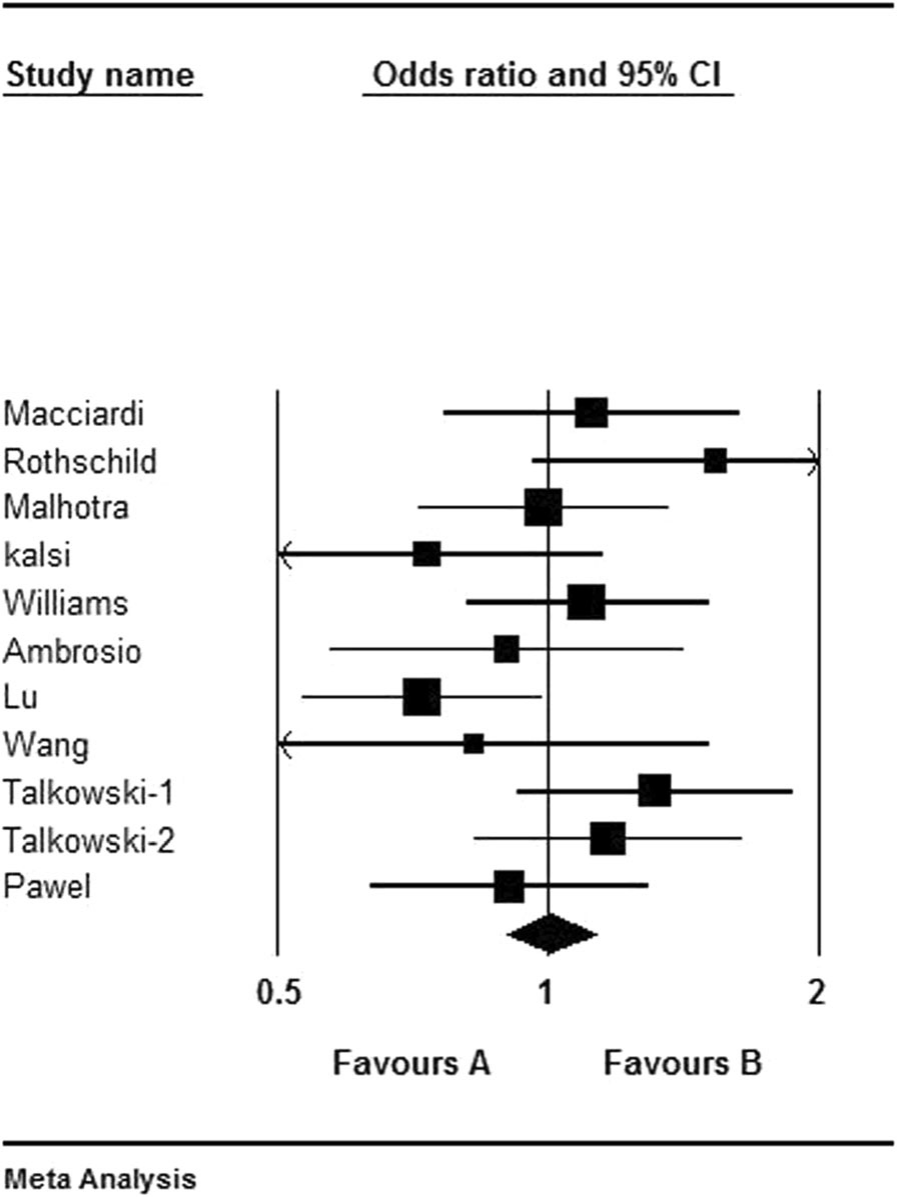
Forest plot of the association between DRD3 Ser9Gly and schizophrenia for TDT studies. **a**: the statistical significance and Ser9 as a protective factor; **b**: the statistical significance and Ser9 as a risk factor

Table 1 showed the pooled ORs and 95% CIs for the 11 original TDT studies with 1219 informative meiosis. There was no statistical significance for the heterogeneity (I^2^ = 28.3%) and the fixed effect model was selected. The pooled results indicated that there were no association between Ser9Gly SNP and schizophrenia (1219 informative meiosis, OR = 1.005, 95% CI = 0.898–1.125, Z-value = 0.086, *p* = 0.932). The forest plot was showed in Fig. 2. Furthermore, we performed the subgroup analysis to further explore the association of Ser9Gly in Caucasian and East Asian populations, respectively. The results indicated the significantly preferential transmission of *DRD3* Ser9 allele in East Asian (204 informative meiosis, OR = 0.744, 95% CI = 0.564–0.980, Z-value = − 2.104, *p* = 0.035), but not in Caucasian (885 informative meiosis, OR = 1.053, 95% CI = 0.923–1.202, Z-value = 0.771, *p* = 0.441). Additionally, the subgroup analysis by diagnostic criteria showed that no association of Ser9Gly was found by DSM-III-R (566 informative meiosis, OR = 1.058, 95% CI = 0.897–1.248, Z-value = 0.673, *p* = 0.501) and DSM-IV (449 informative meiosis, OR = 1.079, 95% CI = 0.897–1.298, Z-value = 0.803, *p* = 0.422). However, there was an association of Ser9Gly by CCMD-III (204 informative meiosis, OR = 0.744, 95% CI = 0.564–0.980, Z-value = − 2.104, *p* = 0.035).

**Table 1:** Meta-analysis of TDT studies of the association between *DRD3* Ser9Gly and schizophrenia

Author	Year	Location	Ethnicity	Diagnostic criteria	Number of transmissions	Ser9 allele		Expected distribution		OR	95% CI	Z-value	***P***-value
						T	NT	T	NT				
Macciardi^41^	1994	Italy	Caucasian	DSM-III-R	108	57	51	54	54	1.118	0.766–1.630	0.577	0.564
Rothschild^42^	1996	USA	Caucasian	DSM-III-R	71	43	28	35.5	35.5	1.536	0.959–2.459	1.786	0.074
Malhotra^43^	1998	USA	Caucasian	DSM-III-R	149	74	75	74.5	74.5	0.987	0.716–1.360	−0.082	0.935
Kalsi^44^	1998	British and Iceland	Caucasian	DSM-III-R	78	33	45	39	39	0.733	0.469–1.146	−1.361	0.173
Williams^26^	1998	Europe	Caucasian	DSM-III-R	160	84	76	80	80	1.105	0.811–1.507	0.633	0.527
Ambrósio^45^	2004	Portugal	Caucasian	DSM-IV	74	35	39	37	37	0.897	0.569–1.416	−0.465	0.642
Lu^46^	2005	China	East Asian	CCMD-III	162	68	94	81	81	0.723	0.531–0.986	−2.047	0.041
Wang^47^	2006	China	East Asian	CCMD-III	42	19	23	21	21	0.826	0.451–1.515	−0.618	0.537
Talkowski^48^	2006	USA	Caucasian	DSM-IV	125	71	54	62.5	62.5	1.315	0.924–1.870	1.523	0.128
Talkowski^48^	2006	India	Indian	DSM-IV	130	70	60	65	65	1.167	0.827–1.646	0.877	0.380
Pawel^49^	2010	Poland	Caucasian	DSM-IV, ICD-10	120	57	63	60	60	0.905	0.632–1.294	−0.548	0.584
Subgroup of East Asian					204	87	117	102	102	0.744	0.564–0.980	−2.104	0.035
Subgroup of Caucasian					885	454	431	442.5	442.5	1.053	0.923–1.202	0.771	0.441
Subgroup of DSM-III-R					566	291	275	283	283	1.058	0.897–1.248	0.673	0.501
Subgroup of DSM-IV					449	233	216	224.5	224.5	1.079	0.897–1.298	0.803	0.422
Subgroup of CCMD-III					204	87	117	102	102	0.744	0.564–0.980	−2.104	0.035
Total					1219	611	608	609.5	609.5	1.005	0.898–1.125	0.086	0.932

Note: T, transmitted (number of times the allele is transmitted from heterozygous parents to the proband); NT, not transmitted

**Fig. 3:**
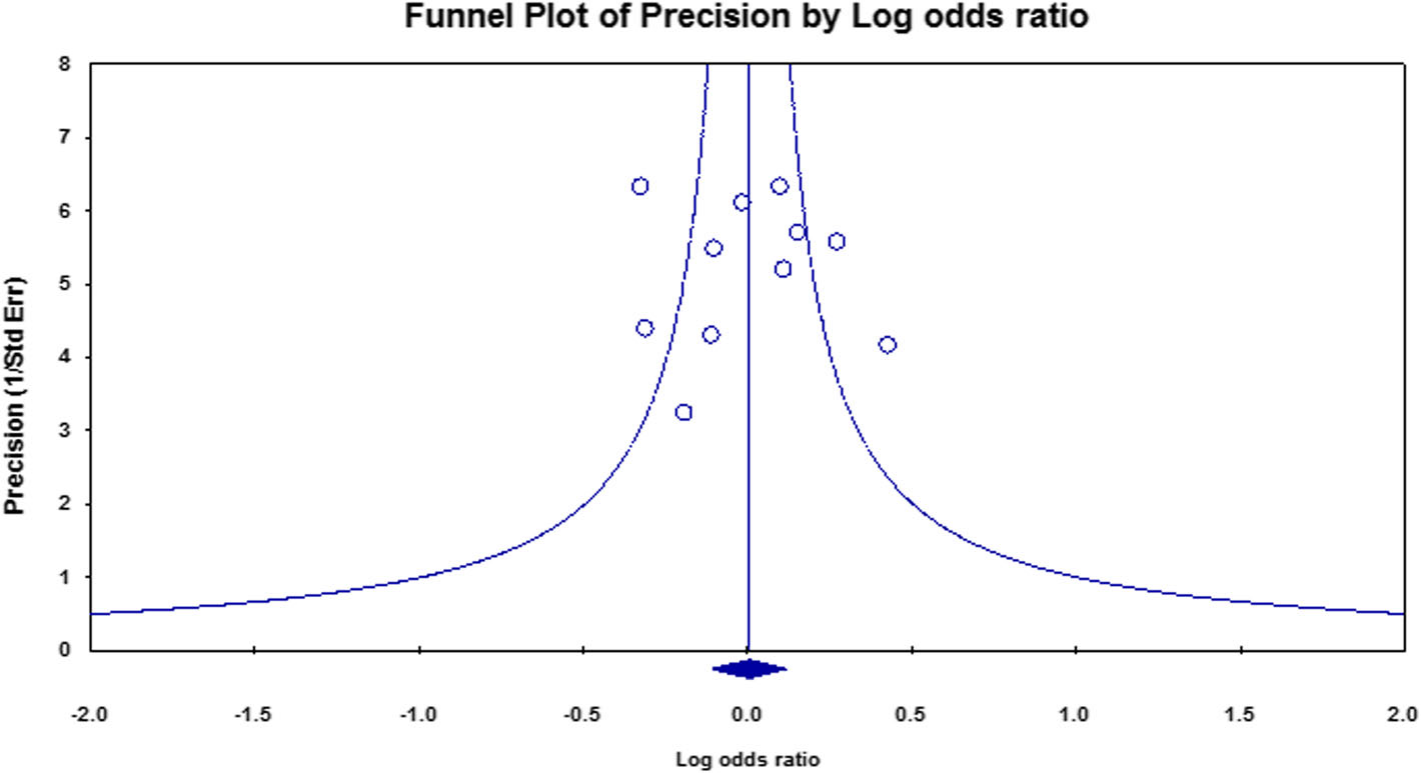
Funnel plot of study precision by log odds ratio for TDT studies

The studies distribution of the funnel plot was substantially symmetrical for the pooled effect size (Fig. 3). Thus, there was not enough evidence for publication bias for TDT studies.

**Fig. 4:**
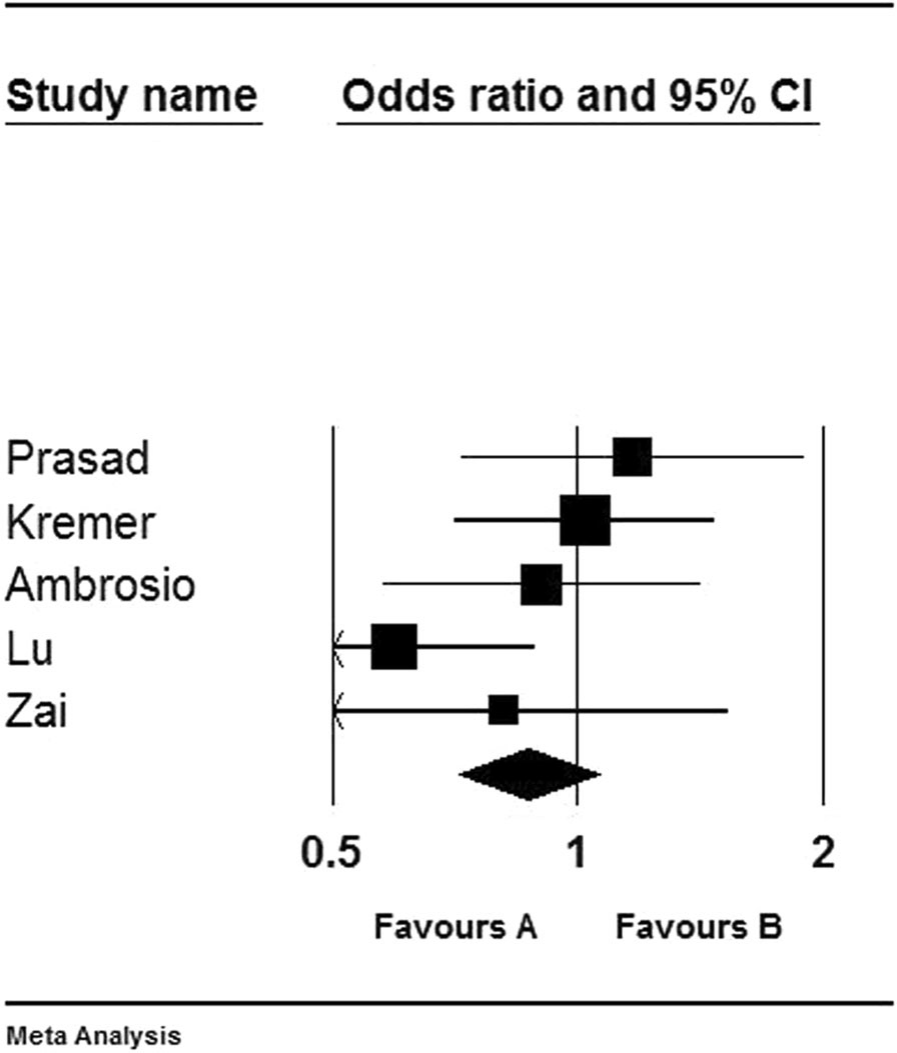
Forest plot of the association between DRD3 Ser9Gly and schizophrenia for HRR studies. **a**: the statistical significance and Ser9 as a protective factor; **b**: the statistical significance and Ser9 as a risk factor

Table 2 showed the pooled ORs and 95% CIs for the 5 HRR studies with 1704 samples. There was no statistical significance for the heterogeneity (I^2^ = 30.372%) and the fixed effect model was selected. The pooled results indicated that there were no association between Ser9Gly SNP and schizophrenia (1704 samples, OR = 0.869, 95% CI = 0.713–1.059, Z-value = − 1.395, *p* = 0.163). The forest plot was showed in Fig. 4. Furthermore, we performed the subgroup analysis to further explore the association of Ser9Gly in Caucasian population. The results indicated no significantly preferential transmission of *DRD3* Ser9 allele in Caucasian (OR = 0.871, 95% CI = 0.604–1.254, Z-value = − 0.744, *p* = 0.457) (Table 3).

**Table 2:** Meta-analysis of HRR studies of the association between *DRD3* Ser9Gly and schizophrenia

Author	Year	Location	Ethnicity	Diagnostic criteria	Sample size	Transmitted		Untransmitted		OR	95% CI	Z-value	***P***-value
						Ser9	Gly9	Ser9	Gly9				
Prasad^47^	1999	India	Indians	DSM-IV	264	67	65	62	70	1.164	0.718–1.886	0.615	0.538
Kremer^48^	2000	Palestinian	Arabian	DSM-IV	516	173	85	172	86	1.018	0.705–1.468	0.094	0.925
Ambrosio^42^	2004	Portugal	Caucasian	DSM-IV	360	122	58	126	54	0.901	0.577–1.409	−0.455	0.649
Lu^43^	2005	China	East Asian	CCMD-III	404	94	108	120	82	0.595	0.401–0.882	−2.584	0.010
Zai^49^	2010	Canada	Caucasian	DSM-IV	160	46	34	50	30	0.812	0.431–1.530	−0.645	0.519
Total					1704	502	350	530	322	0.869	0.713–1.059	−1.395	0.163

**Table 3:** Subgroup analysis of the association between *DRD3* Ser9Gly and schizophrenia in HRR studies

Author	Year	Location	Ethnicity	Diagnostic criteria	Transmitted		Untransmitted		OR	95% CI	Z-value	***P***-value
					Ser9	Gly9	Ser9	Gly9				
Ambrosio^42^	2004	Portugal	Caucasian	DSM-IV	122	58	126	54	0.901	0.577–1.409	−0.455	0.649
Zai^49^	2010	Canada	Caucasian	DSM-IV	46	34	50	30	0.812	0.431–1.530	−0.645	0.519
Total					168	92	176	84	0.871	0.604–1.254	−0.744	0.457

**Fig. 5:**
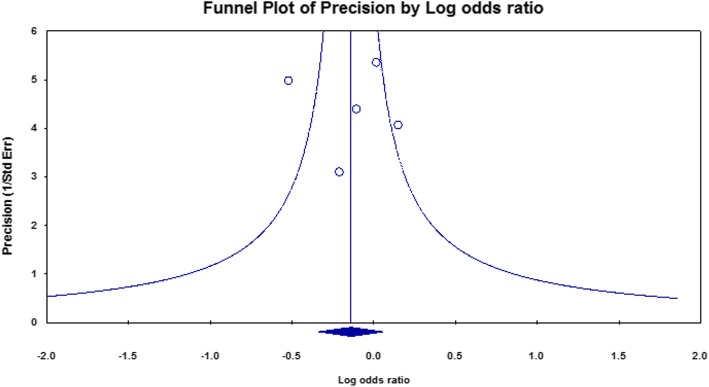
Funnel plot of study precision by log odds ratio for HRR studies

The studies distribution of the funnel plot was slightly asymmetrical for the pooled effect size (Fig. 5). A small but significant effect of publication bias for HRR studies was detected.

## Discussion

We conducted a meta-analysis of family-based association studies (11 for TDT and 5 for HRR) to investigate the putative association of the Ser9Gly SNP in *DRD3* with the risk of schizophrenia. Our overal results suggest that no association exists, except for the significantly preferential transmission of *DRD3* Ser9 allele in East Asian in TDT studies.

Several previous meta-analyses have assessed the potential association of *DRD3* Ser9Gly with the risk of schizophrenia in case-control studies [28, 29, 31–33, 54]. The latest meta-analysis, which included 73 studies comprising 10,634 patients with schizophrenia (cases) and 11,258 controls, suggested that the Ser9Gly SNP is not associated with schizophrenia [33]. Its finding was consistent with our study. Although the subgroup analysis of TDT meta-analysis observed the significant association between Ser9Gly and schizophrenia in East Asian population, it only included two studies with the limited sample size (204 meiosis) [47, 48]. The results of the significantly preferential transmission of DRD3 Ser9 allele in East Asian group showed that the serine allele appears to be protective against schizophrenia. Ser9Gly variant corresponds to a serine to glycine amino acid substitution at position 9 in the extracellular. The substituted glycine allele is thought to yield D3 autoreceptors having a higher affinity for dopamine and more robust intracellular signaling. Subsequently, the increasing DRD3-dependant dopamine intracellular signaling may induce the occurrence of schizophrenia. Moreover, one study of HRR in East Asian also found the significant association, but its sample size was still small (404 samples) [47]. Thus, the positive results need to be interpreted cautiously and more work is required to validate the association in East Asian population. Additionally, it is reasonable that the genetic heterogeneity can lead to the differences in the subgroup analysis of Caucasian and East Asian. Actually, the genetic heterogeneity will complicate the etiology of schizophrenia because the allele distributions of *DRD3* Ser9Gly vary in different ethnicity population. Gly9 allele frequencies vary almost as much in the Japanese control populations (22–34%) as they do in northern and western Caucasian control populations (30–44%) [29, 33]. Therefore, in order to reduce the genetic heterogeneity, it is necessary to study the homogeneous populations.

Presently, numerous candidate genes are involved in the susceptibility of the complex disease, such as schizophrenia. Family-based association studies can provide an informative way to investigate the putative susceptible genes. Unlike population-based tests for association, the family-based tests for transmission disequilibrium are protected against population stratification and the results can avoid the effects of genetic background heterogeneity effectively [55]. Compared with the case-control study with the same sample size, the family-based study is less prone to confounding. Methodologically, it uses a more rigorous approach than the population-based study [56]. Thus, although our previous meta-analysis of case-control studies did not find the significant association of Ser9Gly locus with the risk of schizophrenia, it was still necessary to perform the meta-analysis of family based association.

There were two limitations in our current meta-analysis. Initially, we detected a slight but significant publication bias in the HRR studies. This bias might be due to only English- and Chinese-language studies included. Subordinately, we just evaluated the role of Ser9Gly SNP in the risk of schizophrenia. Nevertheless, only one variation just plays a minute role in the overall genetic susceptibility of the disease. Regrettably, the gene-gene interactions and epigenetics were not assessed without the sufficient information.

## Conclusions

In conclusion, our meta-analysis of family-based association studies found no association between *DRD3* Ser9Gly SNP and the risk of schizophrenia. The large sample homogeneous population studies will be necessary to further explore the role of *DRD3* in the etiology of schizophrenia.

## Data Availability

All data generated or analysed during this study are included in this manuscript.

## Abbreviations

SNP: Single nucleotide polymorphism
DRD3: Dopamine receptor D3
TDT: Transmission disequilibrium test
HRR: Haplotype-based haplotype relative risk
ORs: Odds ratios
CIs: Confidence interval
